# The Stress of Parenting in the Postpartum Period During the COVID-19 Pandemic

**DOI:** 10.1089/whr.2022.0029

**Published:** 2022-11-09

**Authors:** Ka'Derricka M. Davis, Layna Lu, Brittney Williams, Maria V. Roas-Gomez, Karolina Leziak, Jenise Jackson, Joe Feinglass, Lynn M. Yee

**Affiliations:** ^1^ Division of Maternal-Fetal Medicine, Department of Obstetrics and Gynecology, Northwestern University Feinberg School of Medicine, Chicago, Illinois, USA.; ^2^ University of Michigan Medical School, Ann Arbor, Michigan, USA.; ^3^ Division of General Internal Medicine, Department of Medicine, and Department of Preventive Medicine, Northwestern University Feinberg School of Medicine, Chicago, Illinois, USA.

**Keywords:** women's health, parental postpartum challenges, parental challenges, COVID-19, postpartum, postpartum health care

## Abstract

**Background:**

The COVID-19 pandemic produced a major shift in parental roles, which disproportionally exacerbated existing challenges for low-income new parents. Our objective was to identify pandemic-related parenting challenges experienced by low-income postpartum individuals in the context of the early months of the COVID-19 pandemic.

**Methods:**

Semistructured interviews with 40 low-income postpartum individuals were conducted within 10 weeks after giving birth in April 2020–June 2020. Interviews addressed maternal health and well-being, parental stress, including COVID-related barriers to providing for children, and access to essential services. Interview themes were developed using the constant comparative method.

**Results:**

Half (*n* = 20) the participants identified as non-Hispanic Black and 38% (*n* = 15) as Hispanic; 75% (*n* = 30) were parents of multiple children. Parenting-related themes included challenges of parenting multiple children, barriers to maintaining self-care, and novel barriers to providing for children. Participants discussed handling new roles as educators, struggles with entertaining, allocating time among children, and effects of the pandemic on older children. Participants frequently described their lack of alone time, changes in self-care and coping strategies due to continuous parenting, and effects on maternal mental health like increased anxiety. Many participants reported lack of communal support, financial stress, and difficulty accessing services.

**Conclusions:**

New burdens introduced by the pandemic challenged low-income individuals' health and well-being. Understanding these psychosocial stressors and developing interventions to ameliorate these burdens may be key to promoting family health during difficult times; one potential solution for preventing postpartum depression is offering continual social services. Clinical Trial No.: NCT03922334

## Introduction

The postpartum period is a challenging time that requires psychosocial and health care support for postpartum individuals transitioning from pregnancy to parenthood. Nearly 40% of individuals do not attend a postpartum care visit, and the psychosocial stressors that contribute to the development of postpartum depression (PPD) can go unrecognized.^1,2^ In the United States, the prevalence of PPD ranges from 7% to 20% and is impacted by risk factors such as psychosocial stress, low social support, lower socioeconomic status, and major life events or chronic stressors such as death of a loved one and living in a global pandemic during pregnancy.^1,3,4^ Since attendance at postpartum visits is lower, the risk for undiagnosed and untreated PPD is higher among postpartum individuals from disadvantaged populations, including individuals from minority racial-ethnic groups.^1,2,5,6^

Since 2020, the COVID-19 global pandemic has produced a major shift in parental roles, responsibilities, and expectations. Throughout the pandemic, governing bodies worked to prevent the spread of COVID-19 and institutions altered their practices for health care and education, like restricting in-person care, decreasing services provided, and switching to virtual platforms. Recent research has shown the negative effects of the COVID-19 on global stress and anxiety, attributable to experiencing stressors like isolation, inadequate supplies, and financial loss.^7,8^ In a recent study in the United States, parents reported their stress increased, by 50% during the COVID-19 pandemic and as of this year, half remain concerned about COVID-19-related issues such as new variants and returning to remote learning.^9^ Low-income postpartum individuals faced a heavy burden related to caregiving, lack of support, and anxiety about contracting and spreading COVID-19, increasing the risk for PPD.^10–12^

As the toll of the pandemic on pregnancy and postpartum care became apparently clear in April 2020, investigators saw an opportunity for a qualitative investigation of psychosocial stressors affecting underserved patient populations, who are most likely to be heavily impacted by the medical, social, and economic sequelae of the pandemic. Thus, we developed this investigation to develop a better understanding of disruptions that were occurring in both clinical care and social life for newly postpartum parents. The specific objective of this analysis was to identify psychosocial stressors related to parenting, which were experienced by postpartum individuals during the early phase of the COVID-19 in Illinois. Findings will be used to improve postpartum social care for birthing individuals and their families during unusual periods such as pandemics, with an eye to improvements that transcend that specific period and provide general lessons for redesigning postpartum care.

## Methods

In this qualitative study, participants who gave birth between January 2020 and March 2020, at a large (∼12,000 births annually) academic center in Chicago, were interviewed regarding their late pregnancy and early postpartum experiences amidst COVID-19 restrictions and changes. The study interview guide was designed by our multidisciplinary research team, including physicians, patient navigators, and research staff, who have had extensive previous experience with the hospitals' large low-income pregnant population. This diverse team included Black and Latinx individuals with many years of experience of social and medical care for pregnant patients.

The study was designed to evaluate opportunities for future patient navigation capabilities, including services and resources related to parenting, as well as a number of other pandemic-era period patient concerns.^13^ Interview questions focused on participants' experiences of psychosocial stressors related to parenting in the early postpartum period. The questions were designed to elicit conversations about coping strategies that could inform future opportunities for better medical and social care in the context of novel challenges during the pandemic. All methods followed the COREQ guidelines for qualitative research. The study was approved by Northwestern University Institutional Review Board on April 2, 2020, and all participants provided written informed consent.

Eligible participants were individuals who were enrolled in the Navigating New Motherhood study (NNM2), a randomized controlled trial to determine whether patient navigation can improve postpartum health outcomes for low-income individuals (NCT03922334, originally approved April 15, 2019). To be enrolled in the primary study, participants had to be pregnant or postpartum individuals with publicly funded prenatal care and receiving care at one academic medical center in Chicago, Illinois. Participants were 16 years of age or older and English or Spanish speaking. All individuals enrolled in NNM2 at the time of interview conduct were eligible to participate in this interview study.

Individual interviews were conducted over the telephone by three of the authors (M.V.R.-G., K.L., and J.J.), who are trained research assistants, who identify as women, and have experience conducting qualitative research. A semi-structured interview guide with open-ended, in-depth queries was designed to allow participants and interviewers to creatively explore their experiences with health care providers, telemedicine, prenatal care, postpartum care, childcare, and pandemic parenting stress (Table 1 and Supplementary Appendix SA1). This analysis centers on the psychosocial stressors (emotional, social, and financial concerns) of parenting and the postpartum period that arose during this time.

**Table 1.** Selection of Semi-Structured Interview Questions

Prompts and specific questions
First, we will talk about your experience of this epidemic in general, but particularly from the perspective of already being or becoming a mother. Tell us, in your own words, how COVID-19 has affected your experience of pregnancy, parenting, or your anticipation of parenting a new baby. We are interested in all your concerns regarding these issues. How has the current crisis affected your social interactions? How has the current crisis affected your ability to go out of your home? How has the current crisis affected you financially? How has the current crisis affected your mood and emotional well-being? How has the current crisis affected how you perceive your health? Next, we will talk about the experience of COVID-19 as a new mother. How has the current crisis affected your postpartum plans? How has COVID-19 changed your plans to breastfeed? How so/why not? Since you are already parenting a newborn, we are also interested in your parenting experience during this crisis. How has the current crisis affected your ability to buy baby supplies (diapers/formula/etc.)? IF MULTIPLE CHILDREN: How has the current crisis affected childcare for your other children? How has having your other children home affected taking care of your newborn?

Interviews were completed in the participant's preferred language (English or Spanish) and lasted from 30 to 60 minutes. Each interview was digitally audio-recorded and professionally transcribed. Spanish interviews were translated to English by a clinically trained interviewer who was a certified translator. All participants were compensated for their participation.

Transcripts were uploaded on Dedoose (www.dedoose.com), a secure, web-based software application that allows qualitative data management and analysis, by facilitating collaborative data exploration and identification of themes. Transcripts were analyzed by a team of four trained investigators using a constant comparative method that allowed for organization, negation, and agreement on themes and subthemes.^14,15^

The method for the original codebook was detailed in the previous study where four reviewers individually performed an in-depth analysis of an initial subset of transcripts (*n* = 5), mapping emerging concepts. A unified codebook was developed after complete team review to allow for organization, negation, and agreement on themes and subthemes. The codebook was then validated and refined with a second subset of transcripts (*n* = 5), with the reviewers agreeing on eventual saturation of themes. The remaining 30 transcripts were divided and analyzed by two pairs of reviewers, using an ongoing iterative process to ensure consistency with the codebook. Each pair met regularly to ensure agreement on themes and conceptual nodes across reviewers and to resolve discrepancies.

For this analysis, two reviewers organized themes through a thematic analysis and iterative process focusing on psychosocial stressors, including parental mental health and parenting issues. These emerging themes and subthemes are described in the results using exemplary quotations.^13^ In this process, the investigators performed an in-depth review and analysis of emerging concepts related to parental mental health and parenting issues, using a constant comparative method to create a comprehensive codebook with clear operational definitions, which were further developed with subsequent layers of coding.^14,15^ The codebook was then validated and refined with subsequent transcript review, resulting in all coders agreeing on themes and subthemes, as well as saturation of themes. Although the interviews included specific queries about health care access barriers, this analysis centers on the psychosocial stressors (emotional, social, and financial concerns) of parenting and the postpartum period that arose during this time.

## Results

During the interview period (April 2020 to June 2020), there were 50 individuals enrolled in NNM2, of whom 40 (80%) agreed to be interviewed for this study. Interviews took place on average 10 weeks after participants gave birth. Fifty percent of participants identified as non-Hispanic Black and 38% as Hispanic (Table 2). A majority (73%) were parenting multiple children at the time of the interview and 60% were unemployed.

**Table 2.** Participant Demographic Characteristics (*N* = 40)

Characteristic	*N* (%) or mean (SD)^a^
Age, years	28 ± 5
Weeks since delivery	10 ± 3
Race/ethnicity	
Non-Hispanic White	1 (2.5%)
Non-Hispanic Black	20 (50%)
Hispanic	15 (37.5)
Language (interview conducted in)	
English	38 (95%)
Spanish	2 (5%)
Marital status at the time of the interview	
Single/unpartnered	21 (52.5%)
Married/partnered	18 (45%)
Other	1 (2.5%)
Employment at the time of the interview	
Unemployed	24 (60%)
Full-time work	8 (20%)
Part-time/temporary work	6 (15%)
Student	2 (5%)
Parity^b^	
0	11 (27%)
1	13 (33%)
2	4 (10%)
3	6 (15%)
4+	6 (15%)

^a^ Data displayed as *N* (%) or mean ± standard deviation.

^b^ Parity at the time of prenatal recruitment.

The interview responses identifying parental challenges were organized into three major themes: *challenges of parenting multiple children, barriers to maintaining self-care,* and *new barriers to providing for their children*. These themes emerged when participants were asked about their general parenting experience during the crisis, how current crisis has affected the childcare for other children, and how the current crisis has affected their current postpartum plan and ability to provide for themselves and their families. Subthemes were further categorized for specific challenges common to the participants.

### Challenges of parenting multiple children

This theme focused on factors parents of multiple children faced during the early phase of the pandemic. There were four subthemes identified, *new role as an educator, struggles with entertaining, allocating time,* and *effects on older children* (Table 3).

**Table 3.** Challenges of Parenting Multiple Children During Lockdown

Theme	Exemplary quotation
New role as educator (*N* = 14)	Personally, I don't have a lot of patience for that kind of stuff. Like I didn't sign up to be a teacher. I'm not good at explaining things. Like of course I know how to do the work but for me to put it into words without giving answers to her, it can get very frustrating. *—participant 3*
Struggles with entertaining (*N* = 12)	It's like they're stuck at home and it's like what do we do with you guys now? You know it's a little stressful sometimes. Just because we're trying to help them but you know like you don't know what to do and it's just like what do we do now? You know they need stuff to keep them entertained other than their lesson plans or schoolwork or whatnot. So I would say just a little stressed. *—participant 30*
Allocating time (*N* = 12)	At the same time with the new baby, having to juggle between an 8-year-old and her needs and a crying newborn. It's been kinda rough. *—participant 24*
	Totally, yes, totally, because the baby, he needs attention 24 hours a day, he doesn't know, so he is the one that needs the most attention. And the other kids, they also need attention, but it is impossible to give them all the attention that they also want to receive. *—participant 21*
Effects on older child (*N* = 7)	She is absolutely going insane because she likes to play. She wants to be around other children her age, and I don't have any other children, so you know then it seems like she's getting affected all day because we are going through not picking up the baby or putting the baby in danger and she doesn't understand. *—participant 27*

Many parents struggled with the *new role as an educator* when schools introduced virtual learning. Some parents expressed lacking the skills to educate their child(ren) or felt their surroundings prevented them from being successful in creating a suitable learning environment.

One participant stated:

*It's crazy because you know school is out and honestly I don't even know. The teacher is always calling me and I don't know what to tell her. They gave me a tablet and a packet. I don't know what she wants me to do. I can do the more obvious type, the working tablet and logging in and doing all that other stuff is kind of harder for me. I don't even know where to start honestly. I suffer from I have ADD and I have depression, I suffer from depression and anxiety and PTSD. It's just a lot. I don't feel like I can handle the task of being my son's teacher. I just don't, I don't know. I feel like it's too much for me.—participant 23*

Another participant verbalized difficulties with daily communication with the eldest child's teacher, keeping up with the child's school assignments, and feeling constantly pressured about missing assignments:

*I fight with the teacher, who pressures me sending me homework every day, telling me what I need to do and what assignments he has, and sending me and telling me that she has not received any of the assignments. That's what's most frustrating, that it is not only one child, it's four, and sometimes they don't understand that.—participant 21*

In other instances, participants described *struggles with entertaining* their older child(ren), because public parks, entertainment venues, and extracurricular activities were not operating. They were tasked to entertain their child(ren) in unconventional ways for extended periods of time. One parent stated:

*When I do leave my house it's basically running errands. Because I just moved so it's either grabbing things from storage or running errands or sometimes I'll do Instacart just to get the kids out a little bit and they, they like that, they don't have to think about what's going on outside. I mean they still have to be cautious when we're out but they can stop and not have to worry about what's going on. Just sort of like a game of … where's this item type thing, ya know? I try and make it fun for them.—participant 18*

*Allocating time* addressed the participants' difficulties with juggling the needs of a newborn and older children; participants described all children requiring constant attention, while also caring for a newborn. The parents also indicated how having large child age gaps made it difficult since the children needs were so different, one needing assistance with schoolwork and one needing feedings and bonding time. Some participants expressed guilt about their perceived suboptimal parenting.

*I have four kids in total. I have two preschool kids as well and so they were home from school when he [neonate] was discharged from the hospital and the bonding time that I'm able to have with him, I'm not able to really have with him because I'm dedicated like a lot of time, the time that they would be in school they're at home and they're requiring my attention…just everybody needs my attention and it's not really giving me the opportunity to bond with my newborn baby.—participant 20*

About a third of the participants with older children noted the *effects on their older child(ren)'s* emotional and mental health. These participants recounted their older children's frustrations due to lack of mobility and loss of routine.

*They're getting a little sad about it just because you know like we were, we've always been the type of family that always get together over the weekend. So for them not being able to see their cousins or even being at school. I feel like it was just a distraction for them as well and now like they don't really interact with anybody other than dad and I, so they're like we're bored, you know we want this to be over. It's fine, they're coping but yeah, it's just pretty tough on them.—participant 30*

Some participants revealed that their older child(ren) were experiencing increased anxiety around contracting the virus:

*I'm always like the kids are freaking out about every little thing they hear. Like mom they said or you know if like my son was freaking out yesterday, and he has a heart problem, he was saying that he couldn't breathe and that he felt like his throat was hurting. Take a deep breath. Like he really was freaking out about it. I'm like calm down, calm down.—participant 18*

Despite the additional challenges of parenting multiple children during the pandemic, there were few participants who reported a positive experience of having their children at home. Participants highlighted not having to travel for school, enjoying extra help from their older children, spending more time with their older children, and watching their older child bond with the newborn. For example, one participant stated:

*I don't have to drop her off at school, I don't have to pick her up at school, she's amazing and helpful and I'm extremely lucky to have her and then I mean this whole thing's been difficult on her not being with her friends and stuff, but it's actually been without her being in school has been 10 times easier on me. Having her here to help and just even the engagement of having her here I think helps me a lot too.—participant 17*

### Barriers to maintaining self-care

Participants described their *barriers to maintaining self-care* due to the COVID-19 restrictions. Participants emphasized how pandemic changes forced them to “wear multiple hats” and, at times, disrupted the opportunities to take care of themselves. They reported a *lack of free time*, *changes in self-care routine and coping strategies*, *lack of sleep*, and *effects on maternal mental health* (Table 4).

**Table 4.** Barriers to Maintaining Self-Care

Theme	Exemplary quotation
Lack of free time (*N* = 16)	I'll have a friend like come over and kind of give me a break to either take a nap or take my grade school kids like out with their kids…now I am…the mom, the nurse, the mediator, the referee…I'm all of those things, except for help for myself. *—participant 11*
Changes in self-care and coping strategies (*N* = 12)	I love fitness. It's like the one thing in life outside of my kids…the gyms are closed and so it is, I'm sure it's affecting me mentally and physically because I am, I don't have the resources of fitness here, the things that I'm used to…just getting whatever energy or built up things I have inside and so this whole epidemic has stopped that and so it's certainly taking a toll on my weight gain. *—participant 11*
Lack of sleep (*N* = 21)	So that, this has been a very interesting time and it's been pretty stressful and this feels like every day. I'm super fatigued…. I've had like feelings of anxiety and probably because I haven't been able to get much sleep. *—participant 34*
Effects on mental health (*N* = 29)	Emotionally. It's taking a toll on me because I'm very independent or I'm used to getting things on my own and just making things work out so that I can have a smooth lifestyle but sitting in the house, I just, it just gets to me because one, I'm not a homebody. I've cried multiple times because it's just, it messes with my mind sitting in the house all the time and just hearing about how people are dying because of this virus that's going around and the county is just constantly doing it because people just can't sit in their house but it has taken a toll on me emotionally. *—participant 1*

With regard to *lack of free time*, participants reported the inability to “unwind” due to constantly being surrounded by others, including the children, live-in family members, or partners. This burden was attributed to parenting multiple children, having competing roles as partner, parent, educator, and homemaker. A participant described burnout due to a lack of free time:

*It's extremely difficult because those, those down periods award you time to be able to pick up, but if you're overseeing all day you know and don't have a break, then you are overloaded with everything.—participant 27*

In addition, participants discussed not having someone to relieve them and limited time alone. Participants reported having to alter their self-care routines and coping strategies due to having to continuously parent. They expressed burnout and frustration from limited mobility, as well as a perceived reduction in the effectiveness of their coping strategies due to the constant presence of family members and the inability to change their environment.

*I'm a very active and athletic person and being that this is going on, I'm not able to get out and walk or you know go to the gym to do the exercises, so I'm not able to get back to my normal state of physical health.—participant 35*

*Lack of sleep* due to their own family's pandemic anxiety, pandemic restrictions, and having a newborn was reported by more than half of the participants. One participant stated, “I get those panic attacks, so it's hard for me to sleep.” And another stated, “My sleep is affected a little bit because I'm always thinking. Laying in bed you just be thinking about life like when is this all gonna end? What are we gonna do?”*—participant 22*

Fourth, *effects on maternal mental health* address many participant's feelings of depression, anxiety, frustration, and stress resulting from the COVID-19 restrictions. For example, one participant stated:

*My postpartum [depression] was really bad for this pregnancy just because of the pandemic, like being worried and not being able to do everything that I need to do, ya know? It's really hard. Like I think this is the hardest thing that I've had to go through in my life honestly. Because I gave my birth to my 5-year-old daughter and she had a heart defect so she had to have open heart surgery but I never you know went through a pandemic.—participant 23*

Similarly, one participant expressed frustration and increased irritability:

*I mean because of the schools being closed it's definitely frustrating, I would have the time during the day where she would be gone and I'd be able to like relax or take naps or do whatever I need to do but since the schools are closed, we are not able to do that. So, I guess it increases like my irritability a little from being sleep deprived and having an energy-filled almost 7-year-old running around the house.—participant 3*

### New barriers to providing for their children

Almost all participants discussed *new barriers with providing for their children*, namely, the *lack of communal support, financial stress,* and *difficulties accessing services* (Table 5).

**Table 5.** New Barriers to Providing for Their Children

Theme	Exemplary quotation
Lack of communal support (*N* = 10)	They might come and wash dishes for a new mom or come see the baby or wash a load of clothes, something to help. I haven't had anybody…My dad he is close by. He rides his bike past here and waves sometimes, but he can't come in and that's like a big thing…You feel they try to give you support but it's not what it should be because of what's going on. *—participant 27*
Financial stress (*N* = 40)	It gets stressful being at home…not working is stressful. Other than that, the only stress I get from it is like I'm not financially making an income like I usually do. *—participant 13*
	It's kind of, it affected me bad because you know my daughter's father is out of work so he doesn't really help me anymore. So I don't really have that source of help anymore like financially. But like he's there to physically help with my daughter and take care of her but he's out of work and I haven't been working since we found out she was sick, so maybe like 18 weeks pregnant and I just stopped working because it was just too much for me. *—participant 23*
Difficulties accessing services (*N* = 29)	It's a financial concern but also not a lot of stores are necessarily stocked. For instance, I would usually be able to get her milk through WIC, Women and Infant and Children and it was a challenge being able to go and get my WIC coupons to be able to get her milk. *—participant 4*

The *lack of communal support* subtheme includes participants expressing how the COVID-19 exposure risk, societal restrictions, and anxiety decreased the amount of social support expected during the postpartum period, causing burnout and limited social interaction for parents and children (Table 5). For example, one participant stated:

*It's very different to have two children, so I guess the lack of support has made it exponentially more difficult, because I mean it's difficult regardless of how many children you have, but when nobody can really come and help you, yeah, it makes it harder I guess for everyone involved.—participant 3*

The early phase of COVID-19 restrictions caused many businesses to close their doors and causing employees, especially those in lower wage service occupations, to experience *financial stress* (Table 5). Inadequate income was worrisome for participants, while none of participants identified as essential workers, a few had partners and family members who were. Participants had financial concerns even with a temporary moratorium on rent and some bills. One participant revealed needing to ration food to make it last for her household due to a smaller budget, combined with the fear of going to grocery stores:

*It does run out every month. It doesn't last. So, I had to stretch it. Like we have to stretch it as much as I can. I'm trying to limit the food that I make and weigh everything. It's rationing the food that we have.—participant 23*

Despite the distribution of the first stimulus payment in March 2020, participants indicated *financial stress* from the uncertainty of being able to pay bills. The same participant as above also stated:

*I'll think on how much it's weighing down on me. Like I have to worry. Like how am I gonna pay the lights, how am I gonna pay the gas, how am I gonna put food on the table when I run out of the help that I have from the state, ya know? Because it's just, it's, I don't know the word, it's a supplement. It's not a source of income … it's just a supplement, it's just supposed to help.—participant 23*

Third, approximately three-quarters of participants reported *difficulties accessing services* (Table 5). These participants expressed how it was now difficult to receive aid they normally sought out during difficult times, like diaper pantries and social service offices. One participant noted:

*Like other things, yeah, like groceries, like we went to a pantry and they stopped because of the corona. Some people need help and some people just you know really don't need the help. I know some people need a lot of help with all this crisis that's going on.—participant 37*

Similarly, participant 23 also reported problems with other social services:

*I never received her social security card so I won't be able to apply for her disability and get that income that we can have to help get whatever she needs or you know my other children or even food and like it's my daughter is already three months…I've sat on the phone for two hours on hold with the Social Security office and I haven't been able to get through and the offices aren't open to be able to request it.—participant 23*

## Discussion

Our study offers patient perspectives on the novel psychosocial stressors brought on by the COVID-19 pandemic related to new parenting stress during the postpartum period. These perspectives are from a predominantly minority and low-income population, who are more likely to experience increased psychosocial stressors due to structural inequities related to racism and socioeconomic status that increase their risk for adverse health outcomes.^16^

In this analysis, newly postpartum parents faced challenges *of parenting multiple children during lockdown, barriers to maintaining self-care,* and *new barriers to providing for their children*. These included new challenges brought on by the current pandemic-induced crisis and existing challenges that were exacerbated by the crisis. Although this study was conducted in the early phase of the pandemic, our clinical experience of caring for patients over the last 2 years has demonstrated that these themes remain central to the experience of parenting during the pandemic today and raise important issues for social care needs that can reduce the risk of PPD.^9^

### Results in context

There have also been quantitative studies that assess factors that affect parental stress and exhaustion during the lockdown in Europe and the United States, concluding that parents experienced additional stress when there was a lack of communal support during the pandemic, and those who experienced more stress had challenges with being supportive caregivers.^17–21^ Our study offers an initial preliminary examination of the psychosocial stressors by describing other burdens experienced like *financial stress* and *parenting multiple children,* which includes an infant. Another pandemic-period study supported our finding regarding parents and students facing challenges with navigating virtual learning and parental burden.^22^ Unlike our study, it did not offer the unique perspective of a newly postpartum individual, who is both caring for themselves and their family members, as is highlighted in our theme, *new role as an educator*.

Another qualitative study discussed parental experiences during the pandemic, finding that participants reported difficulty with finding coping strategies, loss of support, and effects on their children, which align with our subthemes of *changes in self-care and coping strategies, lack of communal support, and effect on older child mental health.*^23^ Unlike our participants, participants in this prior work discussed recognizing their financial privilege. Similarly, a prior study identified COVID-19 restrictions interrupted access to postpartum social and emotional support,^24^ but did not focus on the unique barriers experienced by low-income individuals. These participants reported more positive experiences like uninterrupted breastfeeding, which contrasts with the financial and social service challenges experienced by participants in our cohort.

Our study corroborates many prior findings, while also specifically identifying that interrupted access to social and emotional support created *new barriers to provide for the children*. Several studies supported the need to monitor worsening pandemic disparities,^16^ and some have proposed solutions that entail providers^25,26^ being supplied with more proactive resources and practical support for worried postpartum parents, like COVID-19 transmission information and breastfeeding support during the COVID-19 pandemic. These perspectives can inform policy and intervention creation that could generate an equitable approach to decrease widening disparities, especially during crises, like a global pandemic.

### Potential solutions

Existing data have suggested parents were less likely to be screened for PPD during the onset of the COVID-19 pandemic.^27^ We suggest multilevel solutions that could diminish the parenting and psychosocial stressors by increasing social support during the COVID-19 pandemic. Additional solutions must occur at the policy level, such as paid maternity leave, which are beyond the scope of this discussion.

Although this study was based on the experiences of parents in an earlier phase of the pandemic, we propose durable changes that are relevant at the current phase of the pandemic and will remain important for supporting young families even past the pandemic's acute effects. Difficulties such as *new barriers to providing for their children* and *lack of communal support* could be addressed by offering emotional and social support virtually or through home care services. Solutions including telemedicine and alternative hybrid options can ensure continuous health and social services access for parenting postpartum individuals in a safe manner.

One study has shown individuals with social support have decreased risk of PPD.^28^ Another study suggested increasing parental support and perceived control over stressful events lowered parental perceived stress and decreased likelihood of child abuse.^18^ As an example of potential future interventions, access to doula services can achieve increased emotional, physical, informational, and social support.^29^ Virtual health care and social services come with its own barriers, including limited access to internet and low digital literacy,^30^ so solutions should also include in-home support, such as from partners, extended family, and postpartum doula services. In United Kingdom, Jackson et al. introduced the idea of postpartum individuals having a “bubble” with a primary support partner, like an extended family member who stays in the home, to comply with pandemic restrictions,^24^ allowing individuals to have some practical, emotional, and mental support.

### Strengths and limitations

The strength of this investigation includes the focus on underrepresented and underserved patients who experienced unique pandemic-related psychosocial stressors related to parenting. In addition, this study is one of the first to provide the perspectives of low-income postpartum individuals in the United States, as the current investigations on this topic have focused on parenting challenges among individuals of a higher socioeconomic status in countries with different health care systems.

However, the sample came from a single urban academic medical center at the beginning of the pandemic, when guidelines were initially being established. Second, interviews did not focus on participants' potential solutions to their challenges, which must be addressed in future work. The identification of psychosocial stressors of parenting and the postpartum period during COVID-19 for low-income individuals is necessary to ensure their perspectives are not ignored during this critical time. Third, some of these topics are common to the postpartum period, so it may be difficult to analyze if it was a new burden due to the postpartum period, the pandemic, or both.

## Conclusion

Although the research was conducted in 2020, its implications and potential solutions can still offer insight to inform policy and intervention creation to help decrease the widening disparity gaps. This study can spark conversation and future work to create sustainable solutions in a world still dealing with the COVID-19 pandemic, especially with the development of new variants and likely enduring social changes that have arisen.

## Supplementary Material

Supplementary Appendix SA1

## Abbreviations Used

NNM2: Navigating New Motherhood study
PPD: postpartum depression
